# The Genetic and Clinical Features of *FOXL2*-Related Blepharophimosis, Ptosis and Epicanthus Inversus Syndrome

**DOI:** 10.3390/genes12030364

**Published:** 2021-03-04

**Authors:** Cécile Méjécase, Chandni Nigam, Mariya Moosajee, John C. Bladen

**Affiliations:** 1 Development Ageing and Disease, UCL Institute of Ophthalmology, London EC1V 9EL, UK; smgxcme@ucl.ac.uk (C.M.); m.moosajee@ucl.ac.uk (M.M.); 2 Kings College Hospital NHS Foundation Trust, London SE5 9RS, UK; chandni.nigam@nhs.net; 3 Moorfields Eye Hospital NHS Foundation Trust, London EC1V 2PD, UK; 4 Great Ormond Street Hospital for Children NHS Foundation Trust, London WC1N 3JH, UK; 5 The Francis Crick Institute, London NW1 1AT, UK

**Keywords:** BPES I, BPES II, blepharophimosis, ptosis, epicanthus inversus, premature ovarian failure, *FOXL2*

## Abstract

Blepharophimosis, ptosis, and epicanthus inversus syndrome (BPES) is a craniofacial disorder caused by heterozygous variants of the forkhead box L2 (*FOXL2*) gene. It shows autosomal dominant inheritance but can also occur sporadically. Depending on the mutation, two phenotypic subtypes have been described, both involving the same craniofacial features: type I, which is associated with premature ovarian failure (POF), and type II, which has no systemic features. The genotype–phenotype correlation is not fully understood, but it has been hypothesised that type I BPES involves more severe loss of function variants spanning the whole gene. Type II BPES has been linked to frameshift mutations that result in elongation of the protein rather than complete loss of function. A mutational hotspot has been identified within the poly-alanine domain, although the exact function of this region is still unknown. However, the BPES subtype cannot be determined genetically, necessitating informed genetic counselling and careful discussion of family planning advice in view of the associated POF particularly as the patient may still be a child. Following puberty, female patients should be referred for ovarian reserve and response assessment. Oculofacial features can be managed with surgical intervention and regular monitoring to prevent amblyopia.

## 1. Introduction

Blepharophimosis, ptosis, and epicanthus inversus syndrome (BPES; OMIM #110100) is a rare autosomal dominant disease, with an estimated prevalence of 1 in 50,000 births, primarily affecting the development of the mid-face structures [1]. The four major clinical signs are dysplasia of the eyelids with shortening of the horizontal fissures (blepharophimosis), droopy upper lids reducing the vertical palpebral aperture (ptosis), bilateral skin fold arising from the medial lower eyelid ascending to the upper lid (epicanthus inversus), and an increased distance between the medial canthi (telecanthus). Two main phenotypes of BPES have emerged, and each harbour the four key ocular signs: (i) type I (BPES-I), which is also associated with premature ovarian failure (POF) involving secondary amenorrhoea before 40 years of age, leading to early menopause and infertility, and (ii) type II (BPES-II) with no systemic associations. 

BPES can be caused by heterozygous variants involving the forkhead box L2 (*FOXL2*) gene, which encodes for a transcription factor expressed predominantly in the developing mesenchyme of eyelids and ovaries [2]. In mice, *Foxl2* expression is localised to the protruding ridges of the developing eyelids and in ovarian follicular cells. Up to 75% of affected individuals may have detectable *FOXL2* mutation, leading to haploinsufficiency [3,4,5,6]. Transmission of BPES-I is usually by affected males as fertility in affected females is reduced due to ovarian dysfunction. BPES-II can have transmission occurring through both males and females. 

In this review, *FOXL2* gene will be detailed with respect to the two types of BPES and the most common variants in the poly-Alanine tract will be reported. Clinical features and management of patients at different stages of life, including referral for ovarian reserve, will be described. 

## 2. Genetics of BPES

### 2.1. FOXL2 Gene

*FOXL2* is a single-exon gene consisting of 2.9 kb (NM_023067.4) located on chromosome 3q22.3. The transcribed protein is 376 amino acids and belongs to the family of forkhead/winged helix transcription factors. FOXL2 regulates a number of genes that control cellular processes including inflammation, transcription, proteolysis, apoptosis, and steroidogenesis including gonadotrophins [7,8]. It is highly conserved among species, with 100% of homology for human, mouse, rat, cow, goat, pig, and rabbit, and consists of a 110 amino acid forkhead DNA-binding domain at position 54 to 148 [9]. It also contains a strictly conserved poly-alanine tract of 14 amino acids between position 221 and 234, whose role remains unknown; however, it is a hotspot for expansions from 14 to 24 alanine residues accounting for ≈30% of all intragenic *FOXL2* pathogenic variants leading to predominantly BPES-II [6,9]. 

Haploinsufficiency of *FOXL2* remains the only reported cause of BPES, and the first autosomal gene implicated in syndromic POF [2]. *FOXL2* can be disrupted by intragenic mutations as well as larger genomic deletions involving the gene locus [10]. More than 250 variants are associated with BPES [11]: intragenic mutations of *FOXL2* account for 81% and can be subdivided into indel frameshift (44%), in-frame deletions (33%), nonsense (12%), missense (11%), and duplications [11]. Whole-gene deletions and larger sub-microscopic deletions encompassing *FOXL2* and neighbouring genes represent 12% and 5% of molecularly confirmed cases, respectively [11]. 

**Figure 1:**
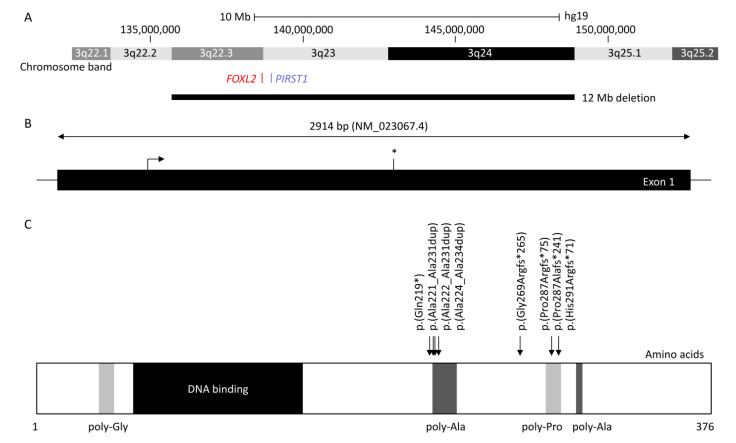
Diagram showing forkhead box L2 (*FOXL2*), up- and downstream genomic position, protein structure, and hotspot located in the poly-alanine domain. (**A**) Chromosomal deletions were reported in at least 84 blepharophimosis, ptosis, and epicanthus inversus syndrome (BPES) patients, with the largest encompassing 3q22.3 to 3q24 (12 Mb) and the smallest encompassing *FOXL2* or *PIRST1* genes (UCSC: hg19:chr3:133,064,629-153,716,375). (**B**) *FOXL2* is composed of one 2.9 kb exon (NM_02367.4). The stop codon is indicated with an asterisk (*). (**C**) FOXL2 is a 376 amino acid protein, composed of poly-glycine (amino acid position 35–43) depicted in light grey, a DNA binding protein or forkhead (amino acid 54–148) in dark, two poly-alanine (poly-Ala) region (amino acid 221–234 and 301–304) in grey, and a poly-proline region (amino acid 284–292) in light grey (Uniprot: P58012). The most common variants (more than five patients were reported) are represented herein, which affect the highly conserved poly-alanine domain (amino acid 221–234) with c.672_701dup p.(Ala224_Ala234dup) (*n* = 80), c.663_692dup p.(Ala221_Ala231dup) (*n* = 12), and c.664_693dup p.(Ala222_Ala231) (*n* = 5), or the poly-proline domain (amino acid 284–292) with c.843_859dup p.(Pro287Argfs*241) (*n* = 46), c.855_871del p.(Pro287Alafs*241) (*n* = 5), and c.855_871dup p.(His291Argfs*71) (*n* = 15). The nonsense variant c.655C>T p.(Gln219*) and the frameshift variant c.804dup p.(Gly269Argfs*265) were reported in 6 and 10 patients, respectively.

At least 460 patients have been reported with *FOXL2*-related BPES [1,6,10,12,13,14,15,16,17,18,19,20,21,22,23,24,25,26,27,28,29,30,31,32,33,34,35,36,37,38,39,40,41,42,43,44,45,46,47,48,49,50,51,52,53,54,55,56,57,58,59,60,61,62]. The most common variants affect two intragenic regions (Figure 1): (i) the poly-alanine region with c.663_692dup p.(Ala221_Ala231dup) reported in 12 patients (four BPES-II cases and eight with undefined type) [6,14,16,30,31,32], c.664_693dup p.(Ala222_Ala231dup) reported in five patients (two BPES-II, two BPES-I, and one with undefined type), and c.672_701dup p.(Ala225_Ala234dup), which was reported in at least 80 patients (24 BPES-II, 2 BPES-I, and 54 with undefined type) [6,14,16,22,26,30,31,32,54,61,63], and (ii) the poly-proline region, which encompasses amino acids at positions 284 to 292, has two duplications variants: c.843_859dup p.(Pro287Argfs*75) reported in 46 patients (3 BPES-I, 2 BPES-II, and 41 with undefined type) [6,14,16,31,32,63] and c.855_871dup p.(His291Argfs*71) in 15 patients (3 BPES-I and 12 undefined type) [6,14,16,24,38,63], and the deletion c.855_871del p.(Pro287Alafs*71) in 5 patients (3 BPES-I, 1 BPES-II, and 1 with undefined type) [6,14,34,40,63]. 

A classification (groups A-H) of intragenic variants in ≈500 *FOXL2* cases was previously proposed by De Baere et al. in order to determine a genotype–phenotype correlation [6]. However, as ovarian function was largely unavailable due to the young age or sex of the patient, this was not possible. A tendency was observed with variants leading to an expanded poly-alanine region, such as c.663_692dup p.(Ala221_Ala231dup), c.664_693dup p.(Ala222_Ala231dup), and c.672_701dup p.(Ala225_Ala234dup), which are mostly associated with BPES-II [6,14,16,22,26,30,31,32,43,45,51,54,61,63]. Interfamilial variability was observed for these variants, where some cases are reported to be associated with BPES-I. Of note, one family reported a BPES-II-affected mother and her BPES-I-affected daughter, both carrying c.822C>G p.(Tyr274*), revealing possible intrafamilial phenotypic variability [6]. 

### 2.2. Intragenic Variants in FOXL2

As *FOXL2* is composed of one exon, it may be resistant to nonsense mediated decay (NMD) as in other cases [64]. Thus, null variants in *FOXL2* will result in a truncated protein leading to partial or complete loss of the forkhead domain and poly-alanine tract [6] or in a shorter protein without the N-terminal region, due to a re-initiation of translation, as observed in COS-7 cell lines transfected with the c.157C>T p.(Gln53*) construct [65]. Whereas duplications within or downstream of the forkhead domain can be predicted to result in an extended protein, mutations involving only part of the forkhead domain may give rise to haploinsufficiency and BPES-II by reducing the transactivation activity of the gene without affecting its DNA binding [66]. The effect of missense mutations can vary depending on their location, which may be in a functionally important region as the gene is highly conserved [66]. Most are mapped to the forkhead DNA binding domain, and these are likely to be pathogenic. However, as missense mutations have been reported in both BPES-I and -II, no prediction can be made regarding the genotype–phenotype correlation [6]. 

### 2.3. Poly-Alanine Tract Expansion Variants

Studies have confirmed the existence of a mutational hotspot in the poly-alanine tract of *FOXL2* in families of different ethnicities [6,67]. This highly conserved region consists of 14 Alanine residues, and the secondary protein structure is predicted to be an α helix, which may become distorted when mutated and disrupt an essential function [6]. Poly-alanine tract expansion is the most common mutation to have been described in BPES-II [67]. Eight different alanine tract expansions (c.663_692dup30 p.(Ala221_Ala231dup), c.664_693dup30 p.(Ala222_Ala231dup), c.664_701dup p.(Ala222_Ala234dup), c.667_702dup p.(Ala223_Ala234), c.672_701dup30 p.(Ala225_Ala234dup), c.684_698dup p.(Ala228_Ala232dup), c.684_698trip15 p.(Ala228_Ala232trip), c.696_728dup p.(Ala232_Ala243dup)) have been described, with the most common consisting of a repeat of 30 bases [6,14,16,22,24,30,31,32,41,54,55,61,63]. These expansions may be caused by slippage of DNA polymerase when duplicating trinucleotide repeats, accounting for about 33% of all intragenic mutations in BPES overall [6,11,41,67]. Whilst expansions are more likely to be associated with BPES-II (without ovarian involvement) and truncated proteins are correlated with BPES-I (with ovarian involvement) [68], some poly-alanine expansions such as c.664_693dup30 p.(Ala222_Ala231dup) and c.672_701dup p.(Ala224_Ala234dup) have resulted in some degree of ovarian dysfunction [14,45]. There has only been one autosomal recessive consanguineous Indian family with evidence of a homozygous poly-alanine tract expansion, c.684_698dup p.(Ala228_Ala232dup), with ovarian failure; segregated carrier parents and siblings were not affected. As such, BPES is mainly regarded as an autosomal dominant disorder [41]. 

Further functional analyses have been performed to study mutation consequences at a cellular level. Luciferase assays revealed some nonsense variants, such as p.(Glu19*), produced a shorter protein with an alternative initiation codon and formed nuclear aggregates, while wild-type protein was diffuse in the nucleus [65]. Caburet et al. showed protein mislocalisation from the nucleus to the cytoplasm with mutated FOXL2 poly-alanine tract expansions leading to its cytoplasmic aggregation [69]. Moreover, these poly-alanine tract expansions lead to decreased expression of several genes involved in apoptosis, transcriptional regulation, mediation of inflammation, cholesterol metabolism, and reactive oxygen species detoxification [70]. Two models were suggested to explain BPES phenotype with or without POF: (1) a higher dose of functional FOXL2 might be required to target promoters in the developing eyelid than in ovarian follicular cells, or (2) the number of FOXL2 binding sites in promoters is the same in both tissues, but aggregation and mislocalisation of mutant protein are stronger in the eyelids than ovaries, due to different tissue-specific proteomics [70]. Some missense variants, such as c.931C>T p.(His311Tyr) associated with BPES-I [6] or with undefined type due to the young age of female patients [62], were also reported to affect the expression of targeted genes, such as the steroidogenic acute regulatory gene (*STAR,* OMIM 600617) [62].

### 2.4. Chromosomal Translocations and Involvement of FOXL2 Regulatory Genes 

Translocation breakpoints in chromosome 3 close to the *FOXL2* gene and *FOXL2* regulatory genes such as *PISRT1* in nine patients carrying t(1;3)(p21;q22), t(1;3) associated with a 1.2 Mb deletion in 3q23 upstream of the *FOXL2* transcription unit, t(2;3)(q33;q23), t(3;4)(q23;p15), t(3;7)(q23;q32), t(3;11)(q22.3;q14.1), t(3;15)(q23;q25), t(3;20)(q22;q13), and t(3;21)(q23;q22.1) were shown to be correlated with BPES [6,12,25,48,52,59,71,72]. Only the patient with t(3;11)(q22.3;q14.1) was known to have BPES-I [48]. Although the *FOXL2* gene itself carried no mutation, positional effects are prevalent in human genetic diseases involving transcriptional factors, for example, *PAX6* and *PITX2* genes, which are involved in aniridia and Axenfeld–Rieger syndrome, respectively [73].

Genomic alterations in loci outside of the *FOXL2* region such as deletion of upstream or downstream regulatory regions close to *FOXL2* or *PISTR1* deletion have also been found to account for about 5% of BPES [53]. Larger deletions encompassing 3q22.3-3q24 are associated with an undefined type of BPES, Dandy–Walker malformation, and Wisconsin syndrome [47], whereas smaller deletions of *FOXL2* or *PISTR1* gene lead to just undefined BPES [63]. Upstream regions of *FOXL2* and *PISTR1* genes are highly conserved in goat, mouse, and human [74], and their deletion leads to polled goats due to PIS (polled intersex syndrome) mutation [75]. This model is characterised by cranio-facial defects, female sterility, and XX sex reversal, associated with decreased expression level of *FOXL2* and *PISTR1* in the ovaries [75]. Luciferase assay revealed the identified genomic deletions in loci outside of the *FOXL2* region affect gene expression in ovarian cell lines [21]. Rearrangements, such as large chromosomal deletion or translocation occurring in these regions, can dissociate the transcription unit from its regulatory elements, resulting in the same phenotype as intragenic mutations [5]. Total and partial gene deletions as well as microdeletions mapping upstream and downstream of *FOXL2* have been found in cases of sporadic and familial cases of BPES [5]. These deletion points are scattered and lie in transcription factor-binding sites and the goat *PIS* locus requiring further investigation to fully understand the intergenic regulatory elements [5]. Deletions were shown to be conserved between different generations of affected family members, revealing meiotic stability. A number of microdeletions, such as a 197 kb deletion upstream of *FOXL2*, have been correlated with BPES-like disorders associated with microcephaly and intellectual disability [13].

### 2.5. FOXL2 and Primary Ovarian Failure (POF) 

FOXL2 is the earliest known but not the sole regulator of sex differentiation in mammals [76]. It is involved in foetal development as well as maintenance of the mature ovary. In the postnatal ovary, FOXL2 supports follicular growth. Ablation of FOXL2 in mice led to atresia of the oocytes with no maturation of secondary follicles. It has been shown that the STAR protein, which is a marker of granulosa cell differentiation, is a direct target of FOXL2, acting as a repressor of *STAR*. It was concluded that the entire alanine/proline-rich carboxyl terminus is important for the repressor activity of FOXL2 and that truncating variants may preferentially lead to BPES and ovarian dysfunction by accelerated differentiation of granulosa cells and secondary depletion of the primordial follicle pool. The identification of a considerable number of ovarian FOXL2 targets may be essential to reveal more insights into phenotypic effects of *FOXL2* pathogenic variants in the adult ovary. Digenic inheritance might contribute to POF associated with BPES through a synergistic effect of *FOXL2* mutations and other genes involved in ovarian function. This may also explain the apparent pleiotropism of *FOXL2* mutations. 

The phenomena of waxing and waning gonadotrophin levels which suggest infertility may be partially reversible in BPES type I. Varying gonadotrophin levels in female patients with BPES type I may not necessarily meet the diagnostic criteria for POF. POF is commonly defined as the presence of four or more months of secondary amenorrhea, postmenopausal levels of follicle-stimulating hormone (FSH; >40 IU/L) all before the age of 40 years [77]. However, there is no universal definition of POF, and coupled with the large normal variation in ovarian reserve, it can be difficult to make a diagnosis of a POF [78,79]. Moreover, spontaneous pregnancies and pregnancies post-stimulation with gonadotrophins have been reported in individuals with *FOXL2* mutations and in women with POF alone [22,80].

## 3. Clinical Features

**Figure 2:**
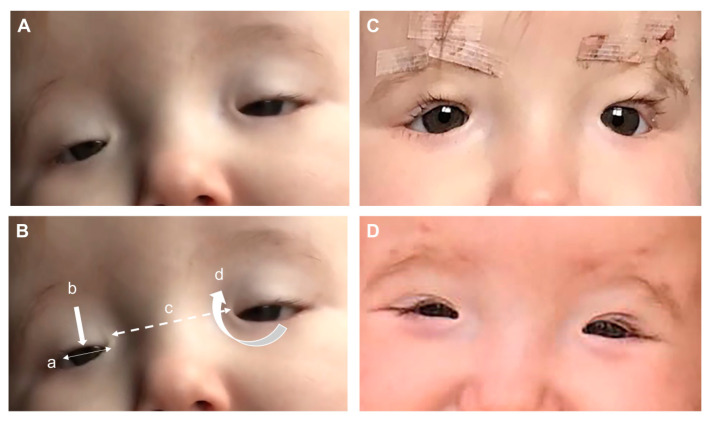
BPES patient with a heterozygous missense mutation in *FOXL2* c.175T>G, p.(Tyr59Asp): (**A**) features seen at 4 months of age consisting of (**B**) four cardinal features: (a) blepharophimosis, (b) ptosis, (c) telecanthus, and (d) epicanthus inversus. (**C**) Bilateral correction of the ptosis at 12 months of age using a Supramid (artificial material) frontalis suspension technique and (**D**) eyelid position 6 months after the frontalis suspension operation.

BPES is mainly a clinical diagnosis based on recognition of the four cardinal signs of bilateral dysplasia of the eyelids with shortening of the horizontal fissures (blepharophimosis), droopy upper lids reducing the vertical palpebral aperture (ptosis), bilateral skin fold arising from the medial lower eyelid ascending to the upper lid (epicanthus inversus), and an increased distance between the medial canthi (telecanthus) at birth (Figure 2). The ptosis is always bilateral, but can be asymmetrical with variability in levator function, but is normally poor [81]. The orbital bones develop normally, and thus the interpupillary distance is usually normal in BPES. Other associated signs, but that are not always present, include turning out of the lower lids (ectropion), lacrimal duct anomalies, strabismus, refractive error amblyopia, broad nasal bridge, thick highly arched brows, short philtrum, and anterverted (low-set) ears [82]. BPES has two phenotypes, with type I associated with premature ovarian failure whereas type II has no associated systemic features. As the genotype–phenotype correlation is unclear, cases of female BPES should be referred to an endocrinologist or fertility specialist. The onset of POF is variable and the diagnosis difficult to make, but an early adolescence referral would be sensible to try and assess ovarian reserve, follicle count, and ovarian response [78,79]. A family history of similar appearance or premature ovarian failure can assist diagnosis. 

### 3.1. Differential Diagnoses

Other congenital disorders may have similar features to BPES, especially two of the cardinal features, blepharophimosis and ptosis. These include hereditary congenital ptosis 1 (OMIM #178300; autosomal dominant, one of three subtypes involving ptosis and blepharophimosis only), OHDO syndrome (OMIM #249620 autosomal dominant with cognitive impairment, congenital heart disease, ptosis, hypoplastic teeth, and blepharophimosis; OMIM #300895 x-linked with coarse facial features, cognitive impairment, and blepharophimosis; and OMIM #603736 autosomal dominant subtype, previously known as Say–Barber–Biesecker–Young–Simpson syndrome), 3MC syndrome (OMIM #257920 autosomal recessive with high arched eyebrows, cognitive impairment, hearing loss, craniosynostosis, hypertelorism, ptosis, and blepharophimosis), Noonan syndrome (OMIM #163950 autosomal dominant with short stature, congenital heart defects, broad forehead, down slanting palpebral fissures, a high-arched palate, and low-set posteriorly rotated ears and hypertelorism), Marden–Walker syndrome (OMIM #248700 cognitive impairment, motor impairment, micrognathia, high arched palate, cleft palate, low-set ears, kyphoscoliosis, joint contractures, hydrocephalus, and blepharophimosis), Dubowitz syndrome (OMIM #223370 autosomal recessive with microcephaly, variable cognitive ability, ptosis, and blepharophimosis), and Smith–Lemli–Optitz syndrome (OMIM #270400 autosomal recessive with cognitive impairment, microcephaly, hypotonia, male hypospadias, multiple internal organ maldevelopment, and ptosis). Blepharophimosis may be observed in aneuploidies which involve deletion of chromosome 3p. If oculofacial features are present without a clear family history or there is any cognitive impairment, these differentials must be considered. Although de novo mutations in BPES are possible, cognitive impairment is not a feature. Early diagnosis is important to allow for appropriate management of both ocular and systemic concerns in these complex patients. 

### 3.2. Management

The management of BPES requires coordination within a multi-disciplinary team, which includes a paediatric ophthalmologist and oculoplastic surgeon, general paediatrician, paediatric endocrinologist, gynaecologist, clinical geneticist, and genetic counsellor. Examination by a paediatric ophthalmologist for visual acuity, refractive error, strabismus, and further management of any amblyopia is essential. An oculoplastic surgeon may evaluate a strategy for surgical correction of the oculofacial abnormalities in order to maximise visual potential. Genetic testing and counselling should be provided by clinical genetics and a genetic counsellor. Referral of female patients to an endocrinologist or gynaecologist during late childhood, puberty, or early adolescent to assess for any onset of POF by looking at ovarian reserve, follicle count, and ovarian response is recommended. 

### 3.3. Genetic Counselling and Testing

A full family history is taken with a pedigree. In those without a family history, there may be reduced or non-penetrance, variable expressivity, or a de novo sporadic change. Severity and prognosis can differ for family members sharing the same disease causing variant termed intrafamilial variability, something that is particularly relevant for POF and poses a challenge for counselling. This variability may be caused by the effects of the environment, epigenetics, and/or modifier genes [6]. 

Being autosomal-dominant, the patient’s offspring have a 50% risk of inheriting BPES, but as females with type I have limited infertility which reduces their chance of having children, BPES-I is skewed towards male transmission. Parents of a proband with no previous family history should undergo segregation testing of *FOXL2* to delineate whether it is a de novo variant and to establish the risk of having further affected children. It is important to consider non-biological explanations such as alternate paternity or undisclosed adoption [83]. A patient’s sibling has minimum risk of having the disease if neither parents are affected and they are born without any eyelid abnormalities, although germline mosaicism has been documented with BPES [3]. If the parent of the proband is affected, the risk to siblings is 50%. 

Genetic testing can involve different approaches. Cytogenetic testing using microarray-based comparative genomic hybridisation (array-CGH) can be used to detect chromosomal abnormalities or copy number variations (CNVs), which contribute to a significant number of BPES patients. If negative, single-gene screening of *FOXL2* or a targeted gene panel including this gene can be undertaken. However, whole-genome sequencing (WGS) is likely to replace this in the future, as it has the capability of detecting CNVs locating the genomic alteration upstream or downstream of *FOXL2* and variants involving non-coding regulatory elements. 

As mentioned in Section 3.1, several syndromes may have overlapping symptoms of BPES, and therefore genetic testing can help to clarify the diagnosis. Despite having a *FOXL2* molecular diagnosis, the two types of BPES cannot be distinguished until puberty due to the lack of robust genotype–phenotype correlation. The disclosure of future reproductive potential is a delicate issue when children are tested. There is a general consensus that genetic testing for disorders with purely reproductive implications for the child should be delayed until the child is old enough to understand the implications of the test and make their own decision regarding testing [4]. This principle, however, could delay diagnostic clarification and timely ovarian tissue cryopreservation, and thus these factors must be evaluated when deciding on the age to discuss family planning and carrying out further genetic counselling. At present, families should be offered help and advice regarding the possibility that the diagnosis of BPES may have the implication of female infertility and support given when informing their offspring of this [4]. It would be appropriate to offer genetic counselling to young females who are affected including discussion of potential risk to offspring and reproductive options [83].

Family planning options include natural conception, assisted conception, in vitro fertilisation, ovarian tissue harvesting, gamete/embryo donation, pre-implantation genetic diagnosis, adoption, or deciding not to have a child. Collection of primordial follicles for embryo or oocyte cryopreservation is also a possibility. It is possible to prevent gene transmission using preimplantation diagnosis, or post-implantation genetic diagnosis of foetal cells via amniocentesis (during 15 to 18 week of gestation), chorionic villus sampling (10 to 12 weeks of gestation), and newer non-invasive prenatal testing (NIPT), which uses a blood sample from the pregnant mother containing cell-free DNA (cfDNA) from the placenta that carries the DNA of the foetus [84]. Significant advances in genetic therapies are being made, and approaches such as CRISPR-Cas9 gene editing, gene replacement, or use of mutation targeting drugs such as nonsense suppression therapy may be applicable in the future [85,86]. 

### 3.4. Treatment of Oculofacial Features 

Treatment in early infancy and childhood is directed towards ensuring full visual development and preventing amblyopia (both unilateral and bilateral). Over 50% of BPES cases will have some degree of amblyopia, mainly from ptosis and strabismus [81,87]. A third of BPES cases will have a refractive error requiring spectacle use [88]. The most obvious cause is occlusion amblyopia from the upper eyelid, obscuring the visual axis. It is common for the baby to develop a chin-up posture early on to allow light to enter the eye, and this does not mean that the vision is developing poorly. A chin-up posture alone is therefore not an absolute indication for ptosis surgery, and this head position can carry on unnoticed by the patient into adulthood. BPES associated with strabismus may also contribute to the failure of development of binocular single vision, and this may require squint surgery to be corrected [81]. Regular vision assessment is paramount during the whole of the child’s visual development phase, normally up until the age of 8. 

If the eyelid is causing an occlusion amblyopia, then a surgical option should be considered. There are multiple ways of lifting the eyelid, and the method used depends on the child’s age and function of the levator palpebrae superioris. Within the first few weeks of life up until the age of 5 years and in the presence of poor levator function, a frontalis suspension using an artificial material can be used [89]. With a mild or moderate levator function ptosis, an anterior or posterior approach ptosis repair can be used [90]. The anterior approach is often described as a supermax levator advancement and the posterior approach also involves a maximal levator advancement [91]. Over the age of 4 and with a poor levator function, tensor fascia lata (autologous or allogenic) can be used for a more permanent solution, but this may be a considered as part of a staged procedure if the epicanthus inversus and telecanthus are to be addressed, as both structures can alter eyelid height [92]. Nevertheless, as with any congenital ptosis, multiple procedures are often required during a lifetime and should be mentioned during the consent process. 

Cosmetic surgery can also be offered to correct the ptosis, epicanthus inversus, horizontal palpebral aperture, and telecanthus. These operations should be personalised to the patient and the family’s needs. Sporadic cases of BPES often undergo cosmetic surgery early on in childhood if the proband is the only one in the family who has the condition. In contrast, inherited cases with multiple family members may wait for when the child is old enough to make their own decision on the need for any cosmetic correction. There is always the “do nothing” option, and this should always be explained to the family and patient when consenting for any surgical procedure. It is possible that early correction during childhood and subsequent ongoing growth of the orbit may allow for a better outcome compared to carrying out the same procedures as an adult when growth has stopped; however, the literature has not described reconstruction in adult BPES. 

Asymmetry between the two eyes is another indication for cosmetic treatment in an attempt to balance the two sides of the face and reduce unwanted attention. Cosmetic reconstruction commonly involves a two-stage procedure which consists of primary surgery correcting the epicanthus inversus and/or telecanthus at around age 3 to 4 and a subsequent operation for the ptosis approximately 6 to 12 months after the initial reconstruction [92]. Various surgical techniques have been applied in the correction of the epicanthus inversus and telecanthus: Y-V plasty, Roveda procedure, Mustardé double Z plasty, and titanium epicanthoplasty [93,94,95,96,97]. A Mustardé double Z plasty is the correction of the telecanthus by removing subcutaneous tissue plus shortening the medial canthal tendon using a suture fixation just posterior to the medial canthal tendon insertion, providing a good cosmetic outcome and avoiding the need for an implant or wire [92,98]. 

### 3.5. Treatment of Premature Ovarian Failure

Female patients with BPES type I co-inherit infertility with the oculo-facial deformities. These patients can be fertile in their early years with normal secondary sexual characteristics. As mentioned, POF is commonly defined as the presence of four or more months of secondary amenorrhea and postmenopausal levels of FSH (>40 IU/L) all before the age of 40 years, although this definition is not universal [77,78,79]. Detection of high-serum FSH, luteinising (LH), and low oestrogen and progesterone levels can indicate the presence of ovarian failure. Follicle count and response to ovarian stimulation may help to assess the ovarian reserve. Pelvic ultrasound may show a hypoplastic uterus and a bone scan may reveal low bone mineral density. Hormone replacement therapy (HRT) long term can be given to alleviate early menopausal symptoms and prevention of sequelae of oestrogen deficiency such as osteoporosis [99]. Advice to improve bone and cardiovascular health should also be employed. Further studies are required to clarify whether ovarian function can be predicted from genotype. Diagnostic difficulty also arises in non-informative families of probands regarding family history and the prevalence of premature ovarian insufficiency due to other causes in patients with BPES. This emphasises the importance of correct assessment of ovarian function in all female patients with BPES despite their genotype. As such, female patients with BPES should be referred to an endocrinologist and/or gynaecologist to be evaluated for POF, although at what age is difficult to ascertain.

BPES patients with primary ovarian insufficiency may consider egg stimulation and retrieval, in vitro fertilisation, and medically induced ovulation. Banking tissue for a future family prior to POF is a newer option and involves ovarian tissue cryopreservation. The tissue can be reimplanted into the ovary for a natural conception if the correct hormonal milieu exists with a normal uterus; alternatively, the eggs can be retrieved from the ovarian tissue and undergo in vitro fertilisation, and then the embryo is implanted into the patient’s uterus or a surrogate. Female patients also require personal and emotional support to deal with the diagnosis and its impact on their health and relationships [99]. 

## 4. Conclusions

BPES requires a multidisciplinary team not only during childhood to ensure maximum visual potential, but into adulthood with often life-long monitoring. The role of *FOXL2* in the pathogenesis of BPES is well established; however, the genotype–phenotype correlation is unclear, even within the same family pedigree, making the prediction of POF in a female individual difficult. Genetic counselling is paramount, with newer testing methods such as whole-genome sequencing highlighting newer mutations and novel therapeutics being discovered. Long-term regular monitoring of vision from birth to prevent amblyopia with swift treatment of any ptosis crossing the visual axis along with any associated refractive error or strabismus is critical to prevent sight loss. Cosmetic reconstruction is personal to the patient and family, but the timing of any intervention will have some effect on outcome, and if desired, both the epicanthus inversus plus telecanthus can be repaired before the ptosis in a two-staged procedure. Puberty will be an uncertain time for all female patients, and when to discuss family planning options is unclear. This should be tailored to each individual family; however, with newer techniques, the ability to have children is greater than ever before.
